# Correction of Class III Malocclusion Treated with Carriere® Motion™

**DOI:** 10.1155/2023/8848581

**Published:** 2023-10-07

**Authors:** Alberto Gentile, Donatella Ferrara, Marta Maci, Domenico Ciavarella

**Affiliations:** Department of Clinical and Experimental Medicine, University of Foggia, 71122 Foggia, Italy

## Abstract

Class III malocclusion needs complex orthodontic treatment. This case report describes a 16-year-old male patient with skeletal class III malocclusion with a negative overjet and overbite. Upper incisors were proclined with the accentuated curve of Wilson. Treatment has changed the functional curve of Wilson that has improved functional dynamic occlusion. The patient was treated using a Carriere® Motion™ Class III (CM3) and SLX 3D Brackets system. After a 25-month treatment, the patient reached class I molars and canines relationships on both sides with good facial aesthetics and good functional occlusion. The result was also satisfactory for the patient. A one-year follow-up confirmed that the outcome was stable.

## 1. Introduction

Class III malocclusion is one of the most complex classes to manage as it affects not only the jaws but the whole craniofacial complex; that is the reason why diagnosis, prognosis, and treatment are always a challenge for orthodontists [1]. Class III malocclusion occurs with both skeletal and dental issues. Skeletal alterations include mandibular hyperplasia, maxillary hypoplasia, or a mix of both. Regarding dental alterations, this class of malocclusion often results in a proclination of the maxillary incisors and retroclination of the mandibular incisors, which then end in a dentoalveolar compensation [2, 3]. About the choice of treatment, it is essential to evaluate many factors such as the main demand of the patient, the age of the patient, the severity of the malocclusion, clinical features, and cephalometric analysis [2].

Growth modification should begin before pubertal growth, after which only orthodontic camouflage or orthognathic surgery are possible choices of treatment [3]. A recently developed approach to class III treatment is the Carriere® Motion™ Class III (CM3) appliance, introduced in 2015 by Luis Carriere of Barcelona, Spain. The CM3 appliance is a modification of the Carriere® Motion™ Class II (CM2) appliance; it is made of bilateral bars bonded to the mesial side of the lower canines and first molars. Flat-molded pads are attached to the center of clinical molar crowns to facilitate mandibular molar distalization. The placement of heavy intermaxillary class III elastics is between hooks that extend anteriorly from bars on the mandibular canines to hooks or buttons attached to the most distal upper molars; elastics are used by the patient as full-time as possible. For anchorage, a clear, invisible upper retainer is used [4].

## 2. Materials and Methods

### 2.1. Diagnosis and Etiology

A 16-year-old male patient presented to us because he did not like his smile and complained he could not chew. The general medical history did not reveal any remarkable information, and there was no previous history of trauma. Pretreatment facial photographs (Figure 1) showed a well-proportioned and symmetrical face, without a gummy smile and with competent lips. The facial profile revealed a straight profile with reduced malar projection; this is a common finding in class III patients with maxillary hypoplasia. There were no TMJ symptoms. No functional jaw movement in close or open movement and modification of CO/OR were evaluated. The intraoral photograph showed a permanent full set of teeth with bilateral molar class III and bilateral canine class I, an anterior crossbite, and an open bite. Both the maxillary and mandibular dental midlines were centered. Oral hygiene was good, and there was no periodontal inflammation.

The pretreatment lateral cephalometric analysis (Figure 2) suggests a class III skeletal pattern (ANB, -1.8°; Wits appraisal, 0 mm; SNB, 88.4°) and a hypodivergent facial pattern (SNMP, 28°). There was a dental compensation that could be seen with upper incisor proclination (U1-SN, 120°), while lower incisors had no alterations in their inclinations (L1-MP, 90.8°). A panoramic radiograph revealed no teeth anomalies and showed the presence of all dental elements except for the third molars that are impacted.

### 2.2. Treatment Objectives

The primary aim of treatment was to correct class III malocclusion and establish a dental and skeletal class I relationship with a good overjet and overbite. The additional objective was to improve facial harmony and aesthetics, acting on the profile as well.

### 2.3. Treatment Alternatives

The first treatment option was an orthodontic treatment with conventional fixed orthodontic therapy that would have taken a lot of time for patients with brackets worn. Orthodontic treatment with a fixed appliance and maxillofacial surgery at the end of growth time was the second option suggested to the patient. Considering the patient's requirement and his adolescent age, the camouflage orthodontic treatment approach using Carriere® Motion™ Class III in the initial period of treatment, followed by a fixed therapy with the SLX 3D Brackets system, was chosen. Extractions were not considered in this case.

### 2.4. Treatment Progress

The first step of the treatment was the application of the Carriere® Motion™ Class III motion appliance to improve the sagittal relationship and achieve a class I by distalizing each mandibular posterior segment, from canine to molar, as a unit. In this stage, we utilized the upper Essix as anchorage and hooked the upper six molars to the lower canines and force 1 elastic (6 Oz 1/4).  Figure 3 shows the case after one month of treatment by Carriere® Motion™ Class III appliance. After three months (Figure 4), when molar class I was almost achieved, we moved on to the second stage; the maxillary arch was bonded with Carriere® SLX .022 “preadjusted, .022” edgewise molar tubes in conjunction with thermally activated Cu Nitanium^∗^ wires (Figure 5). Leveling and alignment started using a nickel-titanium continuous arch wire of 0.014 using light class III elastic tractions (3 Oz 3/16) from the upper molars to the motion hooks located on the lower canines. After one month alignment, continued with a nickel-titanium arch wire of 0.016. Two months later, the Carriere® Motion™ is disassembled, and direct attachments of self-ligating SLX 3D Carriere® are placed on the lower arch with a nickel-titanium (Niti) 0.014 arch wire, always using class III elastics (2 Oz 3/16) (Figure 6). Next month, 0.014 × 0.25 nickel-titanium arch wire was used for the upper arch, and 0.014 nickel-titanium arch wire was used for the inferior arch, replaced the following month by a nickel-titanium 0.016 arch wire for both arches. Arch 016 × 025 upper Cu Niti and Arch 0.018 lower Niti were placed after two months. Next, a lower Arch 0.014 × 0.025 class III elastic bands (6 Oz 1/4) were used. Three months later, following midline evaluation, class II elastics on the right and class III elastics on the left were started to be used to control the midline (3 Oz 3/16). Later, elastic bands were used only at night following the use of the sequence of arc wires: 0.016 × 0.25 upper and 0.014 × 0.25 inferior, 0.018 × 0.25 Cu Niti superior and 0.017 × 0.25 inferior Niti, and 0.018 × 0.25 upper and 0.018 × 0.25 inferior Niti. After one year and a few months of treatment, steel arches were used: 0.018 × 0.25 for both arches (Figure 7). One month later, a 0.019 × 0.25 steel arc was used, always for both arches. During the early stages of treatment, Carriere® Motion™ generates a temporary open bite (Figures 3, 4, and 5) which in the later stages is resolved by fixed orthodontic treatment that results in increased transverse diameters of the arches (Figure 6). After one year of treatment, the molar and canine relationship was achieved (Figure 7). At the end of treatment, Essix removable retainers were delivered to secure the stability of the arches. The total treatment time was twenty-five months (Figure 8).

## 3. Results

Treatment time was twenty-five months; the goals of treatment were reached, the facial profile improved, and the dental relationship reached a molar and canine class I with an ideal overjet and overbite with crowding correction. Dental midlines were centered with the facial midline. Overjet and overbite were normalized (Figure 9). Cephalometric analysis posttreatment showed an improvement of starting parameters (Table 1) (Figures 10 and 11); ANB angle value went from an initial value of -1.7° to -1.2°, thus not improving significantly; a worsening has been avoided; the upper incisors reduced their vestibular position (U1-SN, from 120° to 118°). A mandibular ante rotation is evident by the reduced SNMP value (SNMP from 29° to 26°). Concerning the Wits appraisal, there were no changes after treatment. The complete stability of results one year after the end of treatment is a valuable indicator of future stability (Figure 12).

## 4. Discussion

Angle III classes are definitely one of the most complicated situations for an orthodontist to handle mostly because of the unpredictable and potentially adverse nature of mandibular growth [1–7]. The etiology is multifactorial and complex due to the interaction of hereditary and environmental factors [6]. The class III gold standard treatment is using orthopedic devices in early deciduous or mixed dentition in stage CS2 of cervical vertebral maturation stage [1, 8, 9]. In no severe adult Class III malocclusion, the use of a fixed appliance with class III elastics with bicuspid extractions in both upper and lower arches is a treatment option [10, 11]. In severe cases, the first-choice treatment is maxillofacial surgery to improve functional tooth contacts and the aesthetic facial impact of patients, although many patients do not agree with this type of choice [12, 13]. A recent approach to class III treatment may be the use of the Carriere Motion III in adults or minimally growing patients. The use of the Carriere® Motion™ Class III is a useful and beneficial tool in the nonsurgical treatment of class III that offers an alternative to more complicated therapies [4]. The goal of this device is not only distalization but also the implementation of both skeletal and dental changes, with soft tissue improvement as well. As skeletal changes in fact, this device promotes functional repositioning of the condyle in the temporomandibular complex by the intrusion of the lower molars and extrusion of the lower canine. An important improvement also occurs in the profile, as a result of distalization, retraction of the lower incisors, and a slight advancement of the upper incisors without side effects, reducing what is the classic prognathic appearance of these subjects. In the present case, too, there was an improvement in skeletal values, bringing the ANB value from -1.7° to -1.2°. It also achieved a good overjet and overbite which initially had a value of 0; indeed, at the end of treatment, they are, respectively, 2.5 mm and 1.8 mm. Alignment, molar, and canine first class were evaluated without placing the upper incisors in the vestibular position (U1-SN from 120° to 118°), reaching a good occlusal intercuspation. Upper and lower midlines were centered with the facial midline. The aesthetic profile of the patient has also improved a lot by reducing the prognathic aspect typical of class III patients. CM3, combined with class III elastics and a fixed appliance, is a really useful alternative treatment to correct class III malocclusion. After 6 months of treatment, class correction was reached, and a fixed appliance was introduced at a later stage, leaving the patient without brackets for the first few months while working toward molar class improvement; these are definitely advantages. Compliance with patience is critical to reaching objectives, and the use of an elastic band is essential for the therapy. CM3 determined many changes that were mainly dentoalveolar in nature, but some skeletal changes also occurred, as we have previously seen on cephalometric analysis. Our study agrees with the literature, although there is very little of it. The study by McNamara et al. describes the same findings as the case report here: CMC3 followed by fixed appliances is definitely an excellent alternative effective in treating class III in adult patients, even though the effects are dentoalveolar and minimally skeletal [14].

## 5. Conclusions

In this case report, skeletal class III malocclusion was successfully treated by the Carriere® Motion™ Class III appliance. One-year follow-up showed how results remain stable; this could ensure the stability of this treatment procedure over time. This study shows how the Carriere® Motion™ appliance can be a successful treatment not only in class II but also in class III.

## Figures and Tables

**Figure 1:**
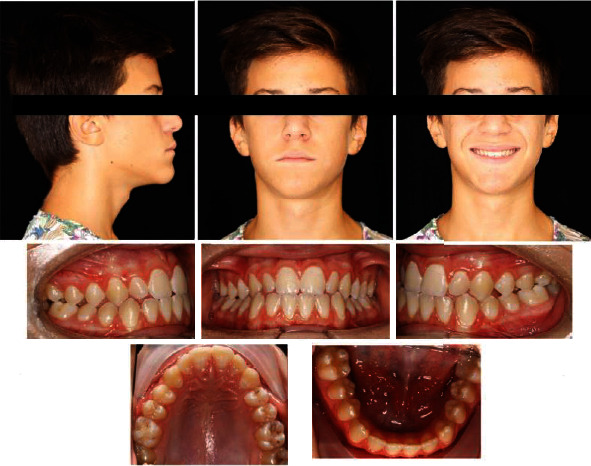
Pretreatment facial and intraoral photographs.

**Figure 2:**
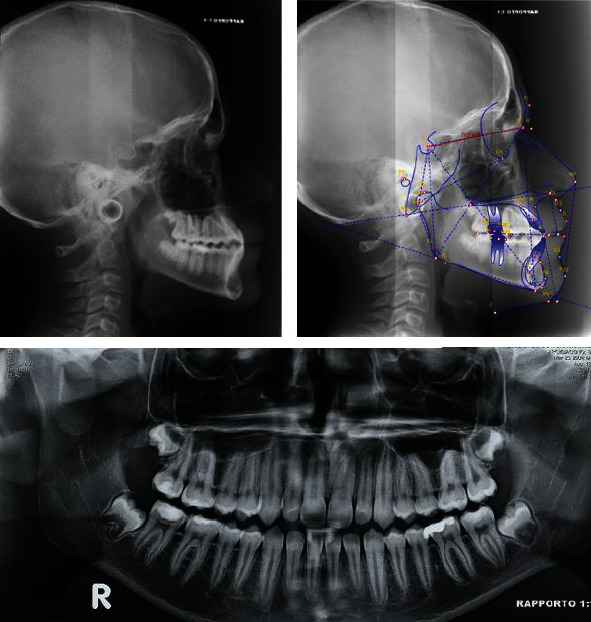
Pretreatment lateral cephalometric radiograph, tracing, and panoramic radiograph.

**Figure 3:**
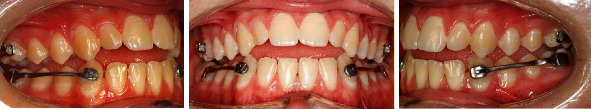
Intraoral photographs of patient after one month of treatment with Carriere® Motion™.

**Figure 4:**
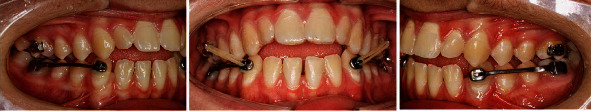
Intraoral photographs of patient after three months of treatment with Carriere® Motion™; upper Essix as anchorage, hook on the upper six.

**Figure 5:**
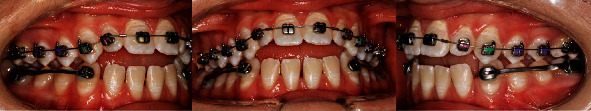
Intraoral photographs of patient after upper bracket application.

**Figure 6:**
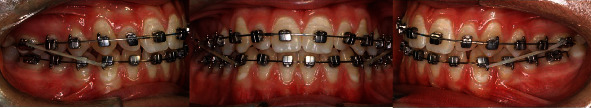
Intraoral photographs of patient after six months of treatment.

**Figure 7:**
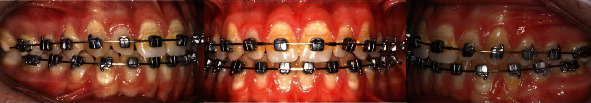
Intraoral photographs of patient after one year of treatment.

**Figure 8:**
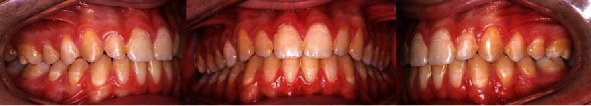
Intraoral photographs of patient after debonding.

**Figure 9:**
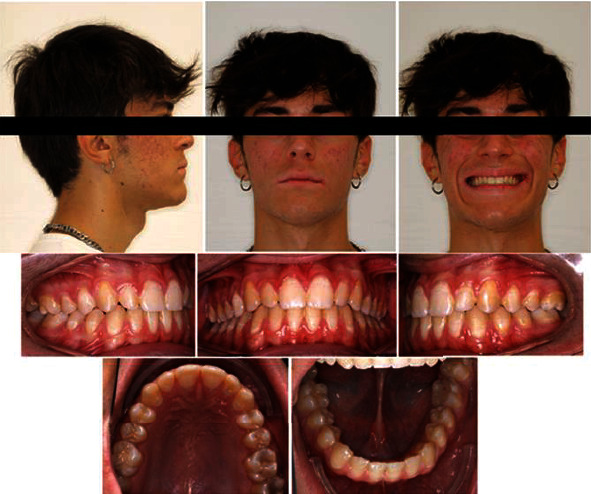
Posttreatment facial and intraoral photographs.

**Figure 10:**
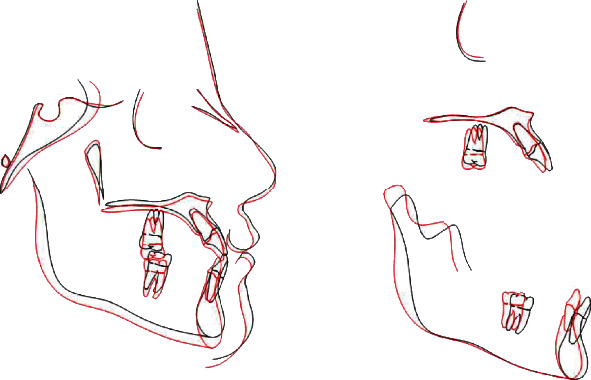
Superimpositions of tracings before and after treatment (black, pretreatment; red, posttreatment).

**Figure 11:**
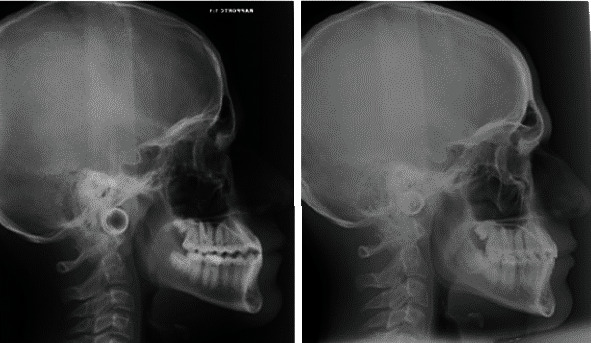
Pretreatment and posttreatment radiographs compared.

**Figure 12:**
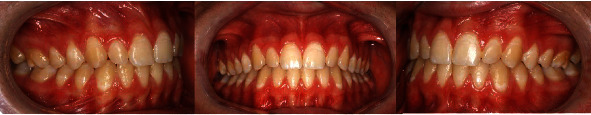
Intraoral photographs of patient one year after debonding.

**Table 1:** Cephalometric analysis.

	Pretreatment	Posttreatment
SNA (°)	85.7	86.6
SNB (°)	87.4	87.8
ANB (°)	-1.7	-1.2
Wits appraisal (mm)	0	0
SNMP (°)	29	26
U1-SN (°)	120	118
U1-facial plane (mm)	3.6	4
L1-MP (°)	90.06	86
L1-facial plane (mm)	3.8	1.2
Overjet (mm)	0	2.5
Overbite (mm)	0	1.8
